# Burying Alive

**Published:** 1874-11

**Authors:** 


### BURYING ALIVE.

While in Munich last year, we drove out to the graveyard. On entering the gateway a crowd of persons were looking through a closed folding door on the left, the upper part being of glass. The first glance showed the greatest profusion of flowers, mostly white. A nearer view presented a large room with a passage in the centre extending from the door to the opposite wall. On either side there were dead persons in their shrouds, of every age, size, sex and condition, extended on boards, the heads about two feet higher than the lower extremities. Some had been dead longer than others, hence were in different stages of decomposition. Some looked ghastly with their great hollow sunken eyes; one, a lady of thirty, seemed to be in a deep sleep; the head was turned a little on one side, towards the door, the face so gentle, so smiling, and so unlike death, with none of its pallor nor wasting away, that it was almost impossible to feel that the body was really dead. There were perhaps fifty in all, some so covered with flowers that only the face was to be seen. A further notice discovered a thin wire from the ceiling extending to each hand, the object being that if any one should come to life again, the slightest motion of the fingers would ring a bell to notify the watchers, who were always near at hand day and night. When a body has remained "twice twenty-four hours" it is removed for burial. On the right hand, also, there were other bodies exposed, protected with glass a foot or two distant from the observer.

It was noticed that no wires were extended to the hands of infants, for the thoughtful German never does a useless thing or goes to an unnecessary trouble or expense. A child of a year old could not know the reason for the attachment of the wire. But really, as a baby might come to life again as well as older persons, and is sometimes worth more than an octogenarian, one would think it ought to have some chance of not being smothered in a close coffin. All babies begin to kick the moment they wake up, and if a wire were attached to their big toes, there would be such a clanking of bell clappers that the drowsiest Dutchman would be waked up in double-quick time.

A physician in his student life, accustomed as he is to see great piles of feet, hands and heads from persons of all ages in the dissecting room, looks at the dead from a scientific standpoint, and is almost entirely relieved of that distressfulness which originally persons experience when confronted with the face of death; hence the sight above was deeply interesting, and awakened suggestions of a varied character; among others, the impression of the kindliness of the custom, as if willing to do all that was possible for the slight chance of returning to life. Then, again, it is a kind of break, an interference with that suddenness between being carried from the house and the terrible thump of the first spadeful of earth on the lowered coffin. It seems as if it would be an easing of this to be able to go night and morning to gaze upon the familiar form, face and features of the loved one who so soon is to be seen no more forever. At the same time it seemed that the crowd looking in upon the flowers and the faces of the dead were merely sight-seers, like ourselves. At all events the whole scene has left pleasant memories; the opening living flowers embowering the forms of death, as typifying the spiritual blooming into immortality and eternal life in the beautiful world above, where death can never enter. Inquiries were made of persons of various ages and stations of life if they had ever known the bell to be rung. No one could remember any such occurrence, nor had a restoration to life ever been heard of within a hundred years. This should allay the fears which some indulge and even cherish of being buried alive, as showing that it is of the rarest occurrence possible. The indications of a living struggle, from change of position of the body which have sometimes been reported, are well nigh impossible of occurrence; and any changes of position are more rationally attributed to the various handlings and joltings of the coffin between the time of the lid being finally closed and the deposit in the vault. Sometimes the coffin is taken down a pair of stairs, sometimes narrow, at others winding, making it necessary to elevate one end or turn it to one side; then there is the jolting of the hearse, and in addition, in lowering into the grave, it seldom happens that it is done horizontally, in consequence of the ropes slipping unevenly in different hands. Perhaps the most appreciable and certain sign of actual death is the appearance of black and blue over the abdomen or stomach. This proves mortification and decomposition. Persons dying of cholera or contagious diseases are buried without delay.

